# Correlation between Overweight, Obesity, Gestational Diabetes Mellitus, Adipokines (Adipolin and Adiponectin), and Adverse Pregnancy Outcomes: A Pilot Study

**DOI:** 10.3390/medicina60091544

**Published:** 2024-09-20

**Authors:** Muntean Mihai, Săsăran Vladut, Luca Sonia-Teodora, Suciu Laura Mihaela, Nyulas Victoria, Muntean Irina Elena, Mărginean Claudiu

**Affiliations:** 1 Departament of Obstetrics and Gynecology 2, University of Medicine Pharmacy Science and Technology George Emil Palade of Târgu Mureș, 540142 Târgu Mureș, Romania; munteanmihai@yahoo.com (M.M.); marginean.claudiu@gmail.com (M.C.); 2 Departament of Neonatology, University of Medicine Pharmacy Science and Technology George Emil Palade of Târgu Mureș, 540142 Târgu Mureș, Romania; lauramihaelasuciu@yahoo.com; 3 Departament of Informatics and Medical Biostatistics, University of Medicine Pharmacy Science and Technology George Emil Palade of Târgu Mureș, 540142 Târgu Mureș, Romania; victoria.rus@umfst.ro; 4 Algcocalm SRL, 540360 Târgu Mureș, Romania; irinagauca@yahoo.com

**Keywords:** adipokines, gestational diabetes mellitus, overweight, obesity, newborn

## Abstract

*Background*: The prevalence of overweight (OW), obesity (OB), and gestational diabetes mellitus (GDM) has been increasing worldwide in recent years. Adipolin is a new adipokine with reduced circulating levels in obesity and type 2 diabetes mellitus (T2DM). *Objectives*: Our prospective case-control study aimed to evaluate the maternal serum levels of adipolin and adiponectin, metabolic parameters, and anthropometric characteristics at the time of oral glucose tolerance test (OGTT) in pregnant women with a pre-pregnancy body mass index (BMI) ≥ 25 Kg/m^2^ and correlate them with newborn adipolin, adiponectin levels, and anthropometric characteristics of the newborns, and secondly to evaluate pregnancy outcomes. *Material and Methods*: After the OGTT results, we had 44 OW/OB pregnant women with GDM, 30 OW/OB pregnant women without GDM, and 92 lean healthy (LH) pregnant women. Data were analyzed by ANOVA and correlation tests, with a *p*-value < 0.05 considered significant. *Results*: We found no differences between adipolin values of the OW/OB pregnant women with GDM and the LH group (*p* > 0.99), OW/OB without GDM and the LH group (*p* = 0.56), and between OW/OB groups (*p* = 0.57). OW/OB pregnant women with GDM had a higher rate of gestational hypertension compared with the LH group (*p* < 0.0001). Newborns from OW/OB pregnant women with GDM were more frequently diagnosed with jaundice (*p* = 0.02), and they required more frequent admission to the neonatal intensive care unit (NICU) for treatment of respiratory distress (*p* = 0.01) compared with newborns from LH mothers. *Conclusions*: Our study revealed that the serum levels of adipolin in the second trimester among the group of OW/OB pregnant women with GDM, matched for age and BMI with OW/OB pregnant women without GDM, were not significantly different. This suggests that adipolin may not play an essential role in the occurrence of GDM in these patients. Despite good glycemic control during pregnancy, OW/OB pregnant women with GDM and their newborns tend to have more complications (gestational hypertension, jaundice, NICU admission) than LH pregnant women and their newborns, highlighting the importance of weight control before pregnancy.

## 1. Introduction

The prevalence of overweight and obesity in Europe has been increasing in recent years, with rates reaching 42.5% for women, and has become a world public health problem. Romania has the highest obesity prevalence among European countries, at 21.1% [1].

In obese individuals, lipotoxicity and low-grade systemic inflammation induce insulin resistance and hyperinsulinemia [2].

In obese pregnant women, in early pregnancy, there is a loss of the reduction in fasting glucose, an increase in insulin resistance in muscle, hepatic, and adipose tissue, and increased lipolysis later in pregnancy [3,4]. These are mediated by the alteration of insulin signaling subclinical inflammation by increasing levels of inflammatory cytokines like tumor necrosis factor-α (TNF-α), IL-6, IL-8, and C reactive protein (CRP), and decreasing adiponectin (an adipokine with insulin-sensitizing properties) and lipogenic transcription factor peroxisome proliferator-activated protein-γ (PPAR-γ) levels [4,5].

Being overweight and obese is linked to increased short- and long-term health risks for both mothers and offspring. Maternal overweight and obesity are associated with increased risk for subfertility, miscarriage, congenital malformations (spina bifida, cleft lip, and palate, hydrocephaly, cardiovascular anomalies), gestational hypertension, preeclampsia, GDM, thromboembolism, intrapartum, and postpartum complications (failed trial of labor, cesarean delivery, post-partum hemorrhage, endometritis) [3,4,6]. Short-term fetal and neonatal complications include increased risk for stillbirth, prematurity, a large for gestational age fetus, macrosomia, shoulder dystocia, and admission to the NICU [3,4,6]. Maternal long-term complications like T2DM and chronic hypertension are related to weight retention after delivery [6]. Neonatal long-term consequences, including increased risk for childhood and adulthood obesity, T2DM, hypertension, neuropsychiatric disorders (autism spectrum disorder), leukemia, and cancer, are mediated through in utero epigenetic modification (DNA methylation), changes in the breast milk composition (higher levels of insulin, leptin), and in maternal and infant gut microbiome [3,4,5].

Anthropometric indices like BMI before pregnancy can be used to define whether a pregnant woman is obese or not [7]. Other anthropometric indices like subscapular skinfold can be used during pregnancy to assess nutritional status [8,9]; other studies did not find correlations between subscapular skinfold thickness and gestational weight gain or newborn weight in overweight or obese pregnant women [10].

GDM is diabetes diagnosed in the second or third trimester of pregnancy that was not diagnosed as overt diabetes before gestation [11]. The prevalence of GDM has increased in recent years, along with the growing prevalence of maternal obesity [12,13]. The highest prevalence of GDM in Europe was found in eastern Europe (31.5%) [14]. Later age at childbearing, high pre-pregnancy BMI, high gestational weight gain (GWG), history of previous macrosomia, GDM in a previous pregnancy, history of diabetes mellitus in first-degree relatives, and subclinical hypothyroidism are described as risk factors for developing GDM [15,16,17]. The pathophysiology of GDM is complex, and in recent years much progress has been made in understanding its mechanism. It includes genetic factors associated with inflammation and insulin resistance in the context of overweight and obesity that unmask β-cell dysfunction. The consequences are hyperglycemia and hyperinsulinemia [18,19]. GDM has short- and long-term complications for mothers and their newborns. Maternal short-term complications include a higher risk for pre-eclampsia, premature rupture of the membrane, induction of labor, and need for cesarean delivery. Fetal and newborn short-term complications include large for gestational age (LGA) and macrosomia, preterm birth, low one-minute APGAR score, respiratory distress syndrome, congenital malformation, and jaundice [20,21]. Mothers with GDM and their infants are at increased risk of long-term complications, including obesity, T2DM, and cardiovascular disease [17].

Adiponectin is a well-known adipokine secreted by adipose tissue. It has insulin-sensitizing and anti-inflammatory effects [22]. The serum level of adiponectin is lowered during pregnancy in healthy, lean women as pregnancy advances through the third trimester [23], and an inverse relation to BMI was observed [24]. The serum level of adiponectin is altered in pregnancy complicated with different pathologies; it is much lower in pregnancy associated with GDM [25,26], obesity [27,28], and preterm labor [29] and higher in pregnancy complicated by preeclampsia [30], suggesting a role of this adipokine in the pathogenesis of these pathologies.

Adipolin, a new adipokine secreted by adipose tissue, has insulin-sensitizing and anti-inflammatory properties and is a paralog of adiponectin [31]. Adipolin or C1q/TNF-related protein 12 (CTRP 12) belongs to the CTRP protein family, like adiponectin. In healthy lean human subjects, hyperinsulinaemic induction significantly increased the circulating levels of adipolin [32]. Its levels are reduced in the adipose tissue and plasma of obese rodents [31] through the upregulation of furin in adipose tissue [33]. Some investigators found that adipolin levels are higher in T2DM patients, probably to modulate insulin resistance, but others found that adipolin levels are lower in T2DM [34,35]. Adipolin was positively associated with fasting plasma glucose, glycated hemoglobin A1c, triglyceride levels, and visceral adiposity index in patients with metabolic syndrome [36]. Adipolin levels were lower in coronary artery disease patients, in which adipolin levels were inversely correlated with BMI and insulin resistance-homeostatic model assessment (IR HOMA), and positively correlated with adiponectin and high-density lipoprotein-cholesterol (HDL-C) levels [37].

There have been no studies until now on adipolin levels in OW or OB pregnant women with or without GDM.

Our prospective case-control study aimed to evaluate the maternal serum levels of adipokines (adipolin and adiponectin), metabolic parameters, and anthropometric characteristics at the time of the OGTT in pregnant women with a pre-pregnancy BMI ≥ 25 Kg/m^2^ and correlate them with newborn adipolin, adiponectin levels, and anthropometric characteristics, and secondly to evaluate the pregnancy outcomes of OW/OB pregnant women with and without GDM compared to each other and with an LH pregnant women group as a control group.

## 2. Material and Methods

### 2.1. Study Design and Subjects

This prospective observational case-control study was conducted in the Obstetrics-Gynecology unit of County Hospital Mures, Romania, from January 2022 to April 2024. During this period, we consecutively enrolled 166 pregnant women, who we further divided according to their BMI into one group of LH pregnant women (BMI = 18.5–24.9 kg/m^2^, n = 92) and a study group of OW/OB (BMI ≥ 25kg/m^2^) pregnant women (n = 74). The study group was further stratified according to oral glucose tolerance test (OGTT) results, made at 24–28 weeks of pregnancy, in two groups, one with OW/OB pregnant women with GDM (n = 44) and the other one with OW/OB pregnant women without GDM (n = 30).

The study group size was chosen based on a priori power analysis with the program G Power Version 3.1.9.6 from Faul et al. [38], using data from Saucedo et al. [39]. Based on these data, we estimated a medium effect size of 0.4, assuming a two-tailed *t*-test with at least 95% power and alpha = 0.05. The total number of 102 patients is the minimum required to sample for sufficient power. We assumed that 166 patients divided into 92 lean, healthy pregnant women, 44 OW/OB women with GDM patients (2:1 ratio), and 30 OW/OB women without GDM (3:1 ratio) would be enough for our study to have sufficient power.

The inclusion criteria were women aged > 18 years with singleton pregnancies, gestational age between 24 and 28 weeks with the OGTT performed, and signed informed consent.

The exclusion criteria were women aged less than 18 years of age, unwilling to sign informed consent, multiple pregnancies, women with type 1 diabetes mellitus or T2DM before pregnancy, fasting plasma glucose of 110–125 mg/dL in the first trimester of pregnancy, inflammatory and vascular disease, hypertension before pregnancy, in utero fetal death, fetal malformations, and use of medications known to affect inflammatory markers.

In all cases, gestational age was estimated by the last menstrual period and ultrasound measurements in the first trimester of pregnancy. All pregnant women were screened for GDM at 24–28 weeks of gestation and classified, according to the screening results and pre-pregnancy BMI, into one of the previously described groups.

Diagnosis of GDM was made according to the International Association of Diabetes and Pregnancy Study Groups criteria by one or more abnormal glucose values during a 75 mg OGTT, with fasting levels ≧ 92 mg/dL (≧5.2 mmol/L), 1 h ≧ 180 mg/dL (≧10 mmol/L), or 2 h ≧ 153 mg/dL (>8.5 mmol/L) [40]. Management after GDM diagnosis was started with nutritional therapy (2000 kcal/day with restriction of carbohydrates to 35%), moderate physical activity (30 min of moderate-intensity aerobic exercise at least five days a week), and subsequent evaluation of glycemic control with fasting glucose and postprandial blood glucose at two hours after meals, daily three times per day for two weeks. Insulin therapy (0.7–1.0 units/kg of body weight daily) was prescribed by a diabetologist (I.E.M) for women who did not achieve glycemic control with diet (fasting glucose levels < 95 mg/dL and postprandial blood glucose values < 120 mg/dL at 2 h) [41]. Thirty-five pregnant women with GDM (79.5%) were treated by diet and moderate physical activity only, and the remainder (n = 9) received insulin therapy for glycemic control. All GDM patients continued to monitor their blood glucose levels until delivery under the surveillance of the diabetologist.

All GDM mothers were seen every two weeks until delivery. OW/OB pregnant women without GDM and LH pregnant women were seen every four weeks until 36 weeks and, after that, every two weeks until delivery. OW/OB pregnant women without GDM received nutritional counseling. They were advised to have moderate physical activity, such as 30 min of walking five times a week, to prevent excessive weight gain during pregnancy.

Upon inclusion in the study, at 24–28 weeks of pregnancy, we collected demographic characteristics like maternal age, history of GDM, family history of diabetes in a first-degree relative, gestations, parity, and educational level through structured questionnaires; we made anthropometric measurements and obtained a fasting blood sample. At delivery, we reviewed the medical records of each patient. We recorded information on pregnancy complications (preterm birth, gestational hypertension, preeclampsia), mode of delivery, anthropometric measurements of the newborns, APGAR score at 1 min, and newborn complications. Also, we obtained an umbilical cord blood sample after clamping the umbilical cord after birth.

Maternal complications were defined as follows:

- Preterm birth = birth at less than 37 completed weeks of gestation, according to ICD 10 definitions (O60).
- Gestational hypertension = systolic blood pressure of 140 mm Hg or more or diastolic blood pressure of 90 mm Hg or more, or both, on two occasions at least 4 h apart after 20 weeks of gestation in a woman with previously normal blood pressure [42].
- Preeclampsia = gestational hypertension and proteinuria 300 mg or more per 24 h urine collection [42].

Neonatal complications were defined as follows:

- Neonatal hypoglycemia = blood glucose level < 36 mg/dL in the first 48 h of age [43].
- Hyperbilirubinemia = neonates who need phototherapy based on their age according to clinical guidelines [44].
- Respiratory distress = need for any form of positive pressure for optimal oxygenation.
- NICU admission = treatment of respiratory distress.

### 2.2. Anthropometric Measurements

The anthropometric measurements of the mothers included in the study were pre-pregnancy BMI, BMI at 24–28 weeks of pregnancy, GWG until the OGTT, and subscapular and suprailiac skinfold thickness. Maternal pre-pregnancy BMI (kg/m^2^) was calculated from pre-pregnancy medical records or, where it was impossible, from weight and height measured during the first prenatal visit during the first trimester of pregnancy. BMI at 24–28 weeks of pregnancy was measured during the OGTT. The patient’s height (cm) was measured using a wall tape without shoes and estimated to the nearest 1 mm. The patient’s weight was measured using a Beurer PS digital scale (Beurer Gmbh, Ulm, Germany), subtracting 0.5 kg from the weight for the patient’s clothing. Gestational weight gain until the OGTT was obtained by subtracting the pre-pregnancy weight from the weight at the time of the OGTT.

Subscapular and suprailiac skinfold thickness, a proxy for central obesity, were measured with a Harpenden Skinfold Caliper (Baty International, West Sussex, UK) calibrated to the nearest 0.2 mm. The same investigator took all measurements twice, using the mean value.

In all cases, newborn anthropometric measurements (weight, length, head circumference, abdominal circumference, and ponderal index at birth) were assessed in the first 30 min after birth. The newborns’ weight was measured using an electronic baby scale U001-BS (Guangzhou Berrcom Medical Device Co., Ltd., Guangzhou, China), and length was measured with an inextensible tape measure.

Ponderal index was calculated as 100 × [birthweight (g)/length (cm^3^)]. Macrosomia was diagnosed in cases of birth weight ≥ 4000 g [45]. Information about newborn sex and APGAR score at 1 min were obtained from medical records.

### 2.3. Biochemical Analyses

We obtained fasting maternal blood samples during the OGTT, between 8 and 10 a.m., using the classic peripheral blood collection technique before any treatment for GDM was administered. Umbilical cord blood was collected by puncture immediately after clamping of the umbilical cord following birth. Laboratory tests were conducted in all cases within 60 min after collection.

From maternal blood, we assessed at 24–28 weeks the CRP level, fasting glucose level, 1 h glucose level, 2 h glucose level after 75 g of orally administered glucose, insulinemia, glycated hemoglobin A1c (Hb A1c), HOMA IR, total cholesterol (TC), HDL-C, low-density lipoprotein cholesterol (LDL-C), and triglycerides (TG).

C-reactive protein (CRP) values were determined by turbidimetry; values of blood glucose by spectrophotometry; glycosylated hemoglobin by turbidimetry; insulinemia by chemiluminescence; and TC, HDL-C, LDL-C, and TG values by photometry with the Atellica Solution CH 930 device (Siemens Healthcare GmbH, Eschborn, Germany). IR HOMA was estimated according to the formula [fasting insulin (mU/l) × fasting glucose (mmol/L)]/22.5 [46].

We assessed adipokine levels from maternal blood at the time of the OGTT and collected umbilical cord blood via puncture immediately after birth to evaluate neonatal adipokine levels. The blood samples were placed in a serum separator tube and left at room temperature for 30 min to allow the serum to clot. After that, the samples were centrifuged at 6000 revolutions per minute for 4 min at room temperature. The serum was then separated and stored at −20 °C until assayed.

Adiponectin and adipolin/CTRP 12 were tested by an automated enzyme immunoassay analyzer (DYNEX DSX Automated ELISA System, DYNEX Technologies Inc, Chantilly, VA, USA) using ELISA kits; Human total Adiponectin/ACRP30, PDRP 300, R&D Systems (Bio-techne, Minneapolis, MN, USA) for adiponectin; and Human C1QTNF12 (C1q and Tumor Necrosis Factor Related Protein 12), NBP2-70032, Novus Biologicals (Bio-techne, Centennial, CO, USA) for adipolin, following the manufacturer’s protocol. The intra-assay coefficient of variation for adiponectin was <4.8% and the inter-assay coefficient of variation was <7.0%; for adipolin, the intra-assay coefficient of variation was <6.0% and the inter-assay coefficient of variation was <5.13%. According to the manufacturer, the sensitivities of the assays were 0.246 ng/mL for adiponectin and 46.88 pg/mL for adipolin.

### 2.4. Statistical Analysis

All data were centralized in a Microsoft Excel database, and the statistical analysis was performed using GraphPad Prism Version.6.0. Student *t*-test or an χ^2^ test was used to evaluate differences in continuous outcomes or categorical variables between the two groups. We used ANOVA analysis to compare continuous parameters with Gaussian distribution between the three study groups. The correlation index was calculated using either the Spearman correlation test or the Pearson Correlation test, depending on the normality of the distribution. Additionally, multiple regression analysis was conducted to identify independent factors. The data are presented as mean ± SD; a *p*-value cutoff of less than 0.05 was considered statistically significant.

Our study has been approved by the Ethics Committee of the University of Medicine, Pharmacy, Science and Technology G E Palade Târgu Mureș (decision number: 1557/2022). It was designed and conducted according to the principles of the Declaration of Helsinki (1964).

## 3. Results

Table 1 shows the demographic, anthropometric characteristics and laboratory results at 24–28 weeks of pregnancy. We found no differences between groups regarding maternal age at inclusion in the study. We found that OW/OB pregnant women with GDM had a significant history of T2DM in first-degree family members and a personal history of GDM, compared with the LH group. Regarding parity, OW/OB pregnant women without GDM had significantly more births than LH women (*p* = 0.03), but there are no differences between OW/OB groups.

We did not find any differences between pre-pregnancy BMI, BMI at the time of inclusion in the study, and GWG until the time of the OGTT among the OW/OB pregnant women groups. Subscapular and suprailiac thicknesses were significantly higher in all OW/OB pregnant groups compared to the LH group (*p* < 0.0001).

Glucose values at the time of the OGTT were significantly higher in OW/OB with GDM compared with LH and OW/OB pregnant women without GDM (*p* < 0.0001). Insulin and IR HOMA values were significantly higher in all OW/OB groups compared with the LH group (*p* < 0.0001). There was no difference between insulin and IR HOMA levels of OW/OB groups (*p* = 0.74 and *p* = 0.14). HbA1c values were higher in OW/OB pregnant women with GDM compared with OW/OB pregnant women without GDM and the LH group (*p* < 0.0001 and *p* = 0.008).

HDL-C levels were lower in the OW/OB with GDM group than in the LH group (*p* = 0.0009), but we did not find any differences between the HDL-C levels of OW/OB groups (*p* = 0.98). TG levels of the OW/OB groups were higher than TG levels of the LH group (*p* = 0.001), but there was no significant difference in TG levels between OW/OB groups (*p* > 0.99). 

There was no difference in the adipolin levels of the OW/OB with GDM group compared with the LH group (*p* > 0.99), the OW/OB without GDM group compared with the LH group (*p* > 0.56), or between OW/OB groups (*p* = 0.57).

Adiponectin values were lower in OW/OB with GDM compared with the LH group (*p* < 0.0001) and in OW/OB without GDM compared with the LH group (*p* < 0.0079), and there were no significant differences between the levels of adiponectin in OW/OB groups (*p* = 0.61).

Table 2 shows the pregnancy outcomes of the mothers included in the study. During the pregnancy, we recorded the following complications: preterm birth, gestational hypertension, and preeclampsia. OW/OB with GDM had a higher rate of gestational hypertension compared with the LH group (*p* < 0.0001). OR for gestational hypertension in OW/OB with GDM compared with LH pregnant women was 26.76 (95% CI 4.16 to 293.6), and in OW/OB without GDM it was 4.11 (95% CI 0.95 to 19.72). There were no differences between groups regarding the rate of preeclampsia. OW/OB pregnant women with GDM gave birth predominantly by cesarean section (68.19%). There were no differences regarding cesarean rates between groups.

Table 3 shows the anthropometric characteristics, adipokine levels, and outcomes among included newborns. Newborns from OW/OB pregnant women with GDM had a more significant weight at birth than the newborns from LH mothers (*p* = 0.04), but there was no difference between the weights of the newborns from the OW/OB mothers group (*p* = 0.85). OW/OB pregnant women with GDM had more macrosomic newborns than LH mothers (*p* = 0.0002). OR for macrosomia in OW/OB pregnant women with GDM compared with LH pregnant women was 13.24 (95% CI, 3.267 to 61.62).

There was no difference between the adipolin newborn levels of newborns from OW/OB mothers with or without GDM (*p* = >0.99) and between newborns from the OW/OB mothers groups and newborns from the LH mothers group.

Newborns from OW/OB pregnant women with GDM had adiponectin levels significantly lower than newborns from LH mothers (*p* < 0.0001) and OW/OB pregnant women without GDM (*p* = 0.01).

Newborns from OW/OB pregnant women with GDM were more frequently diagnosed with jaundice (*p* = 0.02) and required more frequent admission to the NICU for treatment of respiratory distress (*p* = 0.01) compared with newborns from LH mothers. There was no difference regarding newborn outcomes between newborns from OW/OB pregnant women groups.

In correlation analysis, we found a negative correlation between adipolin values at 24–28 weeks of pregnancy and insulin values in OW/OB pregnant women without GDM (r = −0.37). Still, we did not find correlations between anthropometric and metabolic parameters and adipolin levels in OW/OB pregnant women with GDM. In the OW/OB groups, in pregnant women with GDM, adiponectin values were negatively correlated with pre-pregnancy BMI (r = −0.56), BMI at 24–28 weeks of pregnancy (r = −0.61), subscapular thickness (r = −0.64), and HbA1c values (r = −0.66), and positively correlated with HDL-C values (r = 0.15). In OW/OB pregnant women without GDM, adiponectin values were negatively correlated with pre-pregnancy BMI (r = −0.49), BMI at 24–28 weeks of pregnancy (r = −0.47), subscapular and suprailiac thickness (r = −0.41 and r = −0.39), and CRP values (r = −0.41), and positively correlated with TC values (r = 0.55) and LDL-C (r = 0.41). Table 4 shows the correlation between maternal adipolin and adiponectin values at 24–28 weeks with maternal anthropometric, glucose, and lipid homeostasis.

In OW/OB pregnant women with GDM, we found in multiple regression analysis that only BMI at 24–28 weeks is associated with adiponectin levels at 24–28 weeks, and maternal levels of HDL-C and TG at 24–28 weeks of pregnancy are independent variables related to the ponderal index of newborns. Table 5 shows multiple regression analysis for the relationship between maternal adipokines levels, parameters of glucose, and lipid metabolism at 24–28 weeks of pregnancy of OW/OB pregnant women with GDM and maternal and newborn anthropometric characteristics.

## 4. Discussion

Our study aimed to evaluate the maternal serum levels of adipolin, adiponectin, metabolic parameters (fasting glucose, insulin, Hb A1c, HOMA-IR, TC, HDL-C, LDL-C, TG), anthropometric characteristics (pre-pregnancy BMI, BMI at 24–28 weeks of pregnancy, subscapular skinfold thickness, suprailiac skinfold thickness) at the time of the OGTT in pregnant women with a pre-pregnancy BMI ≥ 25 Kg/m^2^ and correlate them with newborn adipolin, adiponectin levels, and newborn anthropometric characteristics, and secondly to evaluate the pregnancy outcomes of OW/OB pregnant women with and without GDM compared with each other and with an LH pregnant women group as a control group.

Adipolin is a new adipokine secreted by adipose tissue; its levels are reduced in the adipose tissue and plasma of obese rodents [31] through the upregulation of furin in adipose tissue [33]. There have been no studies until now on adipolin levels in OW or OB pregnant women with or without GDM.

The serum level of adiponectin is altered in pregnancy complicated by different pathologies; it is much lower in pregnancy associated with GDM [25,26], obesity [27,28], and preterm labor [29], and higher in pregnancy complicated by preeclampsia [30], suggesting a role of this adipokine in the pathogenesis of these pathologies.

There were no statistical differences between the adipolin values of our pregnant women OW/OB groups and the LH control group. Enomoto et al. [47] found that, in diet-induced obese mice, adipolin levels are lower due to the down-regulation of Krúppel-like factor 15 in adipocytes. Tan et al. [48] found that adipolin levels were significantly lower in women with polycystic ovary syndrome (PCOS), which is a proinflammatory state associated with obesity and diabetes. Na et al. [49] found that maternal age and pre-pregnancy BMI were higher, and CTRP 9 (a member of the CTRP family) values in the first trimester of pregnancy were lower in GDM patients compared with the control group, suggesting that CTRP 9 could be an independent risk factor for the occurrence of GDM in pregnancy. Based on our findings, we cannot conclude that adipolin has a role in the occurrence of GDM in OW/OB pregnant women.

The adiponectin values of the OW/OB groups were significantly lower compared with the adiponectin values of the LH group of pregnant women. Adiponectin values between OW/OB pregnant women groups were not different (*p* = 0.61). Hedderson et al. [50] studied adiponectin values before pregnancy and found that women who develop GDM had a pre-pregnancy BMI greater and adiponectin values lower than controls due to altered adipocyte endocrine function in the context of overweight and obesity. Gao et al. [25] and Bao et al. [26] found that adiponectin values were significantly lower in the first and second trimesters of women who develop GDM. Ramirez et al. [51], in a cohort of obese pregnant women of 24–28 weeks of pregnancy with the same pre-pregnancy BMI, found that obese women with GDM had significantly lower adiponectin values compared with obese pregnant women without GDM, suggesting that lower adiponectin values at mid-gestation (in particular high-molecular-weight) together with lower serum values of insulin-like growth factor I binding protein (IGFBP-1) were associated with greater insulin resistance and the development of GDM in these obese pregnant women. Conversely, Vernini et al. [52] and Miturski et al. [53] did not find differences in adiponectin levels among different maternal BMI classes. One explanation of our result regarding the nonsignificant difference between adiponectin values at 24–28 weeks of pregnancy in OW/OB pregnant women with and without GDM is that we included in the study pregnant women with pre-pregnancy BMI ≥ 25 Kg/m^2^, while Ramirez et al. [51] included only obese pregnant women with a BMI ≥ 30 Kg/m^2^, and it is known that adiponectin maternal levels are inversely correlated with maternal BMI [52].

Regarding pregnancy outcomes, we found that OW/OB pregnant women with GDM had a significantly higher rate of gestational hypertension compared with the LH group (*p* < 0.0001). Sibai et al. [54] highlighted in their review that gestational hypertension and GDM are more frequent in obese patients because both are associated with insulin resistance, inflammation, oxidative stress, and vascular disease.

There were more macrosomic newborns in the OW/OB pregnant women with GDM group than in the group of newborns from LH women (*p* = 0.0002). Similarly, Saucedo et al. [39] found that newborns from GDM mothers had a higher weight and ponderal index than newborns from the normal glucose-tolerant pregnant group. Catalano et al. [18] showed in their paper that, in obese pregnant women with GDM, maternal hyperglycemia and hyperlipidemia induce fetal overgrowth. Also, we found in our study group that OW/OB with GDM had higher glucose levels and pronounced dyslipidemia than the LH group.

We found no differences regarding the anthropometric characteristics of the newborns from OW/OB pregnant women with and without GDM. Ramirez et al. [51] found the same results regarding anthropometric newborn characteristics from obese pregnant women with and without GDM. The explanation for this could be that pregnant women with GDM received nutritional and insulin therapy after GDM diagnosis, and OW/OB pregnant women without GDM received nutritional therapy, which ameliorated their metabolic status and prevented overgrowth of the fetuses.

Regarding adipokine levels from the umbilical cord at birth, there were no differences regarding adipolin levels from the umbilical cord between the studied groups or between the adipolin levels of the pregnant women at 24–28 weeks and the adipolin levels from the umbilical cord of the neonates. In contrast with the variation of adiponectin levels from the umbilical cord with birth weight, obesity of the mother, and the presence of GDM, we have no explanation for the adipolin levels in the umbilical cord of the newborns from OW/OB and LH mothers.

Regarding adiponectin levels from the umbilical cord, they are greater than those of their mothers at 24–28 weeks of pregnancy. Also, Sivan et al. [55] found the same difference between maternal and cord blood adiponectin levels. The adiponectin levels of the newborns from OW/OB pregnant women with GDM were significantly lower than adiponectin values from the umbilical cord of the newborns from LH and OW/OB pregnant women without GDM. There are conflicting results in the literature regarding adiponectin levels in the umbilical cord of newborns from GDM mothers compared with newborns from control mothers. Manoharan et al. [56] found lower levels, Ballesteros et al. [57] found no differences, and Aramesh et al. [58] found that adiponectin levels in newborns from GDM mothers are higher than in newborns from normal glucose-tolerant pregnant women. Sivan et al. [55] showed in their article that adiponectin in cord blood is derived from fetal adipose tissue, not from placenta or maternal origins. Our explanation for the lower adiponectin levels in cord blood from the newborns of OW/OB pregnant women with GDM compared with the adiponectin levels from cord blood from newborns from LH mothers stems from the greater weight and adiposity of these newborns and subsequent adipocyte dysfunction.

Regarding newborn complications, newborns from OW/OB pregnant women with GDM were more frequently diagnosed with jaundice (*p* = 0.02), and they required more frequent admission to the NICU for treatment of respiratory distress (*p* = 0.01) compared with newborns from LH mothers. The same results were found by Ye et al. [20] and by us [21] in a cohort of 38 newborns from optimally controlled GDM mothers. The explanation for higher rates of jaundice and respiratory distress could stem from the deleterious effects of hyperglycemia during pregnancy on fetal organ development. We found no difference in the outcomes of newborns from OW/OB pregnant women with GDM compared with the outcomes of newborns from OW/OB pregnant women without GDM.

Regarding the correlation between adipolin values at 24–28 weeks of pregnancy and anthropometric and metabolic parameters in OW/OB pregnant women groups, we only found a negative correlation between adipolin values and insulin values in OW/OB pregnant women without GDM. Compared with the LH group, our OW/OB pregnant women without GDM had greater levels of CRP and insulin and lower levels of adipolin. The insulin resistance state of these patients could lead to lower adipolin levels. Further studies are needed to investigate the adipolin role in OW/OB pregnant women with or without GDM.

In our OW/OB pregnant women with GDM group, adiponectin values were negatively correlated with pre-pregnancy BMI, BMI at 24–28 weeks of pregnancy, subscapular thickness, and HbA1c values, and positively correlated with HDL-C values. Doruk et al. [59] found negative correlations between pre-pregnancy BMI, BMI at the time of the OGTT, HbA1c levels at the time of the OGTT, and adiponectin levels. Kim et al. [60] found negative correlations between adiponectin and maternal BMI at 24–28 weeks of pregnancy. All these findings confirm the effect of overweight, obesity, and associated insulin resistance on adiponectin levels and subsequent GDM occurrence.

In OW/OB pregnant women without GDM, adiponectin values were negatively correlated with pre-pregnancy BMI, BMI at 24–28 weeks of pregnancy, subscapular and suprailiac thickness, and CRP values, and positively correlated with TC and LDL-C values. Our results can be viewed through pregnancy changes, which are characterized by increased insulin resistance and inflammation induced by fat accretion during pregnancy and, consequently, lower adiponectin levels, facts that were also revealed in the article by Catalano et al. [23]. Vernini et al. [52] also found a negative correlation between maternal BMI and adiponectin levels in pregnancy complicated by overweight and obesity. Still, contrary to our study, they did not find a significant difference in adiponectin levels between BMI classes. Poniedziałek-Czajkowska et al. [61] found no significant differences between the adiponectin levels of overweight/obese and normal-weight pregnant women without GDM. They also found a negative correlation between adiponectin levels and BMI and no relationship between adiponectin and CRP values. The differences between our findings and those of Poniedzialek-Czajkowska et al. [61] could be due to the smaller number of overweight and obese patients included in their study.

In multiple regression analysis, we found that only BMI at 24–28 weeks is associated with adiponectin levels at 24–28 weeks in OW/OB pregnant women with GDM. This finding is consistent with the findings of Ramirez et al. [51] and demonstrates that adipocyte function is altered in the context of obesity and insulin resistance. Higher levels of obesity and insulin resistance lead to lower adiponectin levels.

Regarding the relationship between maternal adipokine, carbohydrates, and lipid parameters of OW/OB pregnant women with GDM and newborn anthropometric parameters, we found a relationship between the levels of HDL-C and TG and the newborns’ ponderal index. Barbour et al. [62] found that TG and free fatty acids are strong contributors to excess fetal weight at birth, particularly in obese pregnant women. Also, Olmos et al. [63] found that maternal TG in the second and third trimesters of pregnancy of overweight and obese GDM mothers with good glucose control is partially responsible for LGA newborns. Contrary, Boghossian et al. [64] found minimal associations between second-trimester maternal lipid levels and neonatal anthropometrics, suggesting that glucose levels or other factors may influence newborn anthropometric characteristics. One limitation of the conclusions of Boghossian et al. [64] could be the smaller sample size of GDM patients included in the study. One explanation for increased newborn weight in the OW pregnant women with GDM group comes from the work of Song et al. [65], which showed that placental fuel transport and storage genes were increased in these patients.

What was known: Adiponectin levels are lower in pregnant OW/OB women with GDM compared to LH women at the time of diagnosis; the newborns of mothers with GDM have lower adiponectin values than the newborns of healthy mothers.

What was not known: The adipolin level in the second trimester of OW/OB pregnant women with and without GDM, adipolin levels of the newborns of OW/OB pregnant women with and without GDM.

What is new: The level of serum adipolin in OW/OB pregnant women with GDM at the time of the OGTT does not differ from that of OW/OB pregnant women without GDM and of LH pregnant women. The adipolin cord blood levels of the newborns of OW/OB pregnant women with GDM do not differ from the adipolin levels of newborns from OW/OB pregnant women without GDM or of the newborns of LH mothers. The adiponectin levels at the time of the OGTT of OW/OB pregnant women with GDM are not significantly lower than the adiponectin levels of OW/OB pregnant women without GDM. We cannot conclude that adipolin and adiponectin are significantly involved in the pathogenesis of GDM or can be used as a biomarker of GDM in pregnant women with pregestational overweight or obesity.

The practical implication of our study is that we cannot use adipolin in the second trimester as a predictor of GDM in OW/OB pregnant women.

We want to acknowledge that our study has some limitations: We only measured total adipolin and adiponectin and did not measure their isoforms. There are works such as Ramirez et al. [51] that show the lower level of adiponectin is due to a lower level of high molecular weight isoform of adiponectin. We must mention that we have limited patients in the study group. We assessed adiposity by BMI and subcutaneous fat by subscapular and suprailiac skinfold thickness as a surrogate for central obesity. Still, we did not assess visceral adipose tissue, which is known as a risk factor for T2DM, hypertension, heart attack, angina, and hyperlipidemia [66].

Our study has several strengths: We used groups of OW/OB pregnant women with the same age and BMI and did not use the patients’ self-reported weight and height for BMI calculation. All participants were closely monitored at a single center, and the GDM diagnosis was strictly conducted per specific criteria, ensuring uniformly collected data. We assessed fasting adipokine levels at the time of the OGTT and from umbilical cord blood at birth. This is the first study conducted in Romania to evaluate the level of adipolin in OW or OB pregnant women with and without GDM and their newborns.

## 5. Conclusions

Our study revealed that the serum levels of adipolin in the second trimester among a group of OW/OB pregnant women with GDM, matched for age and BMI with OW/OB pregnant women without GDM, were not significantly different. This suggests that adipolin may not play an essential role in the occurrence of GDM in these patients. We found a negative correlation between adipolin levels and insulin levels in OW/OB pregnant women without GDM. We did not find a significant difference between adiponectin levels in OW/OB with and without GDM at the time of the OGTT, questioning the role of adiponectin in the occurrence of GDM in these patients. There are no differences in adipolin levels of newborns from the OW/OB groups; adiponectin is much lower in the cord blood of newborns from OW/OB mothers with GDM than newborns from OW/OB mothers without GDM. In multiple regression analysis in OW/OB pregnant women with GDM, we found a relationship between BMI at the time of the OGTT and adiponectin levels and between HDL-C and TG maternal levels at the time of the OGTT and newborn ponderal index. Despite good glycemic control during pregnancy, OW/OB pregnant women and their newborns tend to have more complications (gestational hypertension, macrosomia, jaundice, NICU admission) than LH pregnant women and their newborns, highlighting the importance of weight control before pregnancy.

## Figures and Tables

**Table 1:** Demographic, anthropometric characteristics and laboratory results at 24–28 weeks of pregnancy.

Characteristics	Lean Healthy Group (LH)—24–28 Weeks (n = 92)	OW/OB without GDM—24–28 Weeks (n = 30)	OW/OB with GDM—24–28 Weeks (n = 44)	*p*-Value		
				LH vs. OW/OB without GDM	LH vs. OW/OB with GDM	OW/OB without GDM vs. OW/OB with GDM
Age, years	31.05 ± 4.74	30.33 ± 5.32	31.84 ± 4.95	>0.99	0.79	0.49
History of T2DM in first-degree family members	7 (7.52%)	4 (13.33%)	14 (31.81%)	0.46	0.0006	0.09
Personal history of GDM	2 (2.17%)	2 (6.66%)	6 (13.63%)	0.25	0.01	0.46
Education						
9–12 years	26 (28.26%)	5 (16.66%)	13 (29.54%)	0.23	>0.99	0.27
>12 years	66 (71.73%)	23 (76.66%)	27 (61.36%)	0.33	0.24	0.21
Gestational age, weeks	25.67 ± 1.31	25.93 ± 1.25	26.25 ± 1.34	>0.99	0.053	0.95
Gestations	1.82 ± 1.03	2.4 ± 1.03	1.95 ± 0.88	0.008	0.73	0.23
Parity	1.52 ± 0.68	1.86 ± 0.73	1.68 ± 0.70	0.03	0.47	0.74
Pre-pregnancy BMI, kg/m^2^	21.21 ± 1.87	28.47 ± 3.12	30.57 ± 4.98	<0.0001	<0.0001	>0.99
BMI, kg/m^2^	24.57 ± 2.28	31.03 ± 3.17	33.20 ± 4.75	<0.0001	<0.0001	>0.90
GWG until OGTT, kg	8.72 ± 3.68	6.89 ± 4.54	7.04 ± 4.78	0.22	0.09	>0.99
Subscapular thickness, mm	16.78 ± 6.31	23.76 ± 10.28	26.27 ± 8.44	<0.0001	<0.0001	0.79
Suprailiac, mm	17.6 ± 7.18	23.52 ± 10.35	24.17 ± 9.27	0.0002	<0.0001	>0.99
CRP, mg/dL	0.63 ± 0.53	0.90 ± 0.64	0.88 ± 0.74	0.07	0.15	>0.99
Fasting glucose level, mg/dL	79.58 ± 5.38	81.83 ± 5.99	97.68 ± 13.79	0.41	<0.0001	<0.0001
1 h glucose level, mg/dL	123.3 ± 23.54	130.8 ± 24.08	178.5 ± 38.22	0.42	<0.0001	<0.0001
2 h glucose level, mg/dL	102.5 ± 16.89	102.5 ± 23.6	141 ± 38.47	0.99	<0.0001	<0.0001
Insulin, mUI/l	12.47 ± 22.34	20.42 ± 31.61	28.73 ± 58.23	<0.0001	<0.0001	0.74
HbA1c, %	4.89 ± 0.32	4.97 ± 0.34	5.4 ± 0.59	0.32	<0.0001	0.008
IR HOMA	2.42 ± 4.33	4.24 ± 6.82	7.39 ± 15.81	0.0001	<0.0001	0.14
T-cholesterol, mg/dL	249.3 ± 36.95	243.6 ± 40.42	232 ± 49.42	0.78	0.059	0.46
HDL cholesterol, mg/dL	75.96 ± 14.06	68.70 ± 15.36	66.35 ± 17.57	0.12	0.0009	0.98
LDL cholesterol, mg/dL	156.3 ± 36.68	148.1 ± 37.96	139.4 ± 43.62	0.58	0.0501	0.61
Triglycerides, mg/dL	188.3 ± 62.25	244.9 ± 82.81	238.2 ± 88.52	0.001	0.001	>0.99
Adipolin, pg/mL	5402 ± 581.1	5197 ± 706.2	5227 ± 1269	0.56	>0.99	0.57
Adiponectin, ng/mL	6845 ± 2134	5422 ± 2080	4735 ± 1800	0.0079	<0.0001	0.61

Note: Data are presented as means (standard deviation), counts, and percentages. OW/OB = overweight/obese; T2DM = type 2 diabetes mellitus; GDM = gestational diabetes mellitus; BMI = body mass index; GWG = gestational weight gain; OGTT = oral glucose tolerance test; CRP = C reactive protein; HbA1c = glycosylated hemoglobin; IR HOMA = homeostasis model of assessment for insulin resistance; T-cholesterol = total cholesterol; HDL cholesterol = high-density lipoprotein cholesterol; LDL cholesterol = low-density lipoprotein cholesterol.

**Table 2:** Comparison of maternal pregnancy outcomes between the study groups.

Parameters	Lean Healthy Group (LH) (n = 92)	OW/OB without GDM (n = 30)	OW/OB with GDM (n = 44)	*p*-Value		
				LH vs. OW/OB without GDM	LH vs. OW/OB with GDM	OW/OB without GDM vs. OW/OB with GDM
Preterm birth ≦ 37 weeks	6 (6.52%)	2 (6.66%)	6 (13.63%)	>0.99	0.20	0.46
Gestational hypertension	1 (1.08%)	2 (6.66%)	10 (22.72%)	0.14	<0.0001	0.10
Preeclampsia	2 (2.17%)	1 (3.33%)	0	>0.99	>0.99	0.40
Vaginal birth	30 (32.6%)	12 (40%)	14 (31.81%)	0.11	0.42	0.62
Cesarean section	62 (67.4%)	18 (60%)	30 (68.19%)	0.50	>0.99	0.62

Note: Data are presented as counts and percentages. OW/OB = overweight/obese; GDM = gestational diabetes mellitus.

**Table 3:** Anthropometric characteristics, adipokine levels, and outcome among included newborns.

Parameters	Newborns From LH Mothers (n = 92)	Newborns From OW/OB Mothers without GDM (n = 30)	Newborns From OW/OB Mothers with GDM (n = 44)	*p*-Value		
				Newborns From LH Mothers vs. Newborns From OW/OB Mothers without GDM	Newborns From LH Mothers vs. Newborns From OW/OB Mothers with GDM	Newborns From OW/OB Mothers without GDM vs. Newborns From OW/OB Mothers with GDM
Birth weight, g	3303 ± 381.8	3451 ± 549.8	3510 ± 561.1	0.29	0.04	0.85
Birth weight ≧ 4000 g	2 (2.15%)	3 (10%)	10 (22.72%)	0.09	0.0002	0.21
Male	46 (50%)	18 (60%)	20 (45.45%)	0.40	0.71	0.24
Apgar 1 min score, ≦8	5 (5.43%)	1 (3.33%)	3 (6.81%)	>0.99	0.71	0.64
Newborn length, cm	53.05 ± 2.11	53.27 ± 2.33	53.34 ± 2.54	>0.99	>0.99	>0.99
Head circumference, cm	33,73 ± 1.33	34.17 ± 1.55	34.16 ± 1.44	0.77	0.59	>0.99
Abdominal circumference, cm	32.05 ± 1.51	32.18 ± 2.05	32.76 ± 2.37	0.94	0.10	0.39
Ponderal index	2.20 ± 0.22	2.26 ± 0.19	2.29 ± 0.21	0.32	0.06	0.88
Adipolin pg/mL	5580 ± 848.8	5792 ± 776.4	5785 ± 746.3	0.61	0.24	>0.99
Adiponectin ng/mL	35,340 ± 37,464	29,449 ± 23,448	19,842 ± 8070	0.72	<0.0001	0.01
Neonatal hypoglycemia	1 (1.08%)	1 (3.33%)	3 (6.81%)	0.43	0.09	0.64
Jaundice,	21 (22.82%)	9 (30%)	19 (43.18%)	0.46	0.02	0.33
NICU admission (respiratory distress treatment)	1 (1.08%)	2 (6.66%)	5 (11.36%)	0.14	0.01	0.69

Note: Data are presented as means (standard deviation), counts, and percentages. LH = lean healthy, OW/OB = overweight/obese; GDM = gestational diabetes mellitus; NICU = neonatal intensive care unit.

**Table 4:** Correlation between maternal adipolin and adiponectin values at 24–28 weeks with maternal anthropometric, glucose, and lipid homeostasis.

Adipokine		Adipolin		Adiponectin			Adipolin		Adiponectin	
Variables ↓	OW/OB without GDM	Correlation Coefficient—r	*p* Value	Correlation Coefficient—r	*p* Value	OW/OB with GDM	Correlation Coefficient—r	*p* Value	Correlation Coefficient—r	*p* Value
Pre-pregnancy BMI		−0.22	0.22	−0.49	0.006		−0.20	0.18	−0.56	0.03
BMI at 24–28 weeks		−0.16	0.39	−0.47	0.007		−0.25	0.09	−0.61	0.01
Subscapular thickness		−0.23	0.21	−0.41	0.02		−0.21	0.15	−0.64	0.004
Suprailiac Thickness		−0.18	0.32	−0.39	0.03		−0.01	0.93	−0.48	0.17
CRP		−0.19	0.31	−0.41	0.02		−0.20	0.17	−0.48	0.13
Fasting glucose		−0.12	0.52	0.02	0.90		−0.02	0.87	−0.38	0.57
Insulin		−0.37	0.04	−0.25	0.18		0.16	0.29	−0.22	0.13
HbA1c		−0.11	0.55	−0.08	0.65		0.03	0.83	−0.66	0.002
IR HOMA		−0.35	0.053	−0.21	0.25		0.15	0.32	−0.53	0.07
TC		0.23	0.20	0.55	0.001		0.11	0.47	−0.12	0.23
HDL-C		0.17	0.35	0.35	0.057		−0.04	0.76	0.15	0.002
LDL-C		0.14	0.43	0.41	0.02		−0.002	0.98	0.06	0.69
TG		0.02	0.88	−0.09	0.61		0.10	0.50	−018	0.21
adipolin				0.10	0.56				0.03	0.82
adiponectin		0.10	0.56				0.03	0.82		

Note: OW/OB = overweight/obese; GDM = gestational diabetes mellitus; BMI = body mass index; CRP = C reactive protein; HbA1c = glycosylated hemoglobin; IR HOMA = homeostasis model of assessment for insulin resistance; T-cholesterol = total cholesterol; HDL cholesterol = high-density lipoprotein cholesterol; LDL cholesterol = low-density lipoprotein cholesterol; TG = triglycerides.

**Table 5:** Multiple regression analysis, *p*-values, for the relationship between maternal adipokines levels, parameters of glucose and lipid metabolism, and maternal-newborn anthropometric characteristics in OW/OB pregnant women with GDM.

Maternal and Newborn Anthropometric Characteristics	Adipolin *p*-Value	Adiponectin *p*-Value	Insulin Level *p*-Value	IR HOMA *p*-Value	HDL-C *p*-Value	TC *p*-Value	Fasting Glucose *p*-Value
Pre-pregnancy BMI	0.37	0.11	0.68	0.65	0.60	0.92	0.99
BMI at 24–28 weeks	0.20	0.05	0.74	0.71	0.92	0.70	0.97
Birth weight	0.45	0.60	0.44	0.44	0.24	0.42	0.63
Cranian circumference	0.67	0.59	0.86	0.96	0.21	0.24	0.22
Abdominal circumference	0.52	0.66	0.57	0.60	0.74	0.39	0.39
Ponderal index	0.73	0.65	0.97	0.96	0.04	0.05	0.44

Note: BMI = body mass index; IR HOMA = homeostasis model of assessment for insulin resistance; HDL cholesterol = high-density lipoprotein cholesterol; TC = total cholesterol.

## Data Availability

The data supporting this study’s findings are available from the corresponding author (V.S.) upon reasonable request.
